# Assessment of radiation damage behaviour in a large collection of empirically optimized datasets highlights the importance of unmeasured complicating effects

**DOI:** 10.1107/S0909049511008235

**Published:** 2011-04-09

**Authors:** Tobias Krojer, Frank von Delft

**Affiliations:** a Structural Genomics Consortium, University of Oxford, Oxford, UK

**Keywords:** radiation damage, data collection, strategy, beamline software, datasets

## Abstract

The radiation damage behaviour in 43 datasets of 34 different proteins collected over a year was examined, in order to gauge the reliability of decay metrics in practical situations, and to assess how these datasets, optimized only empirically for decay, would have benefited from the precise and automatic prediction of decay now possible with the programs *RADDOSE* [Murray, Garman & Ravelli (2004 ▶). *J. Appl. Cryst.* **37**, 513–522] and *BEST* [Bourenkov & Popov (2010 ▶). *Acta Cryst.* D**66**, 409–419]. The results indicate that in routine practice the diffraction experiment is not yet characterized well enough to support such precise predictions, as these depend fundamentally on three interrelated variables which cannot yet be determined robustly and practically: the flux density distribution of the beam; the exact crystal volume; the sensitivity of the crystal to dose. The former two are not satisfactorily approximated from typical beamline information such as nominal beam size and transmission, or two-dimensional images of the beam and crystal; the discrepancies are particularly marked when using microfocus beams (<20 µm). Empirically monitoring decay with the dataset scaling *B* factor (Bourenkov & Popov, 2010 ▶) appears more robust but is complicated by anisotropic and/or low-resolution diffraction. These observations serve to delineate the challenges, scientific and logistic, that remain to be addressed if tools for managing radiation damage in practical data collection are to be conveniently robust enough to be useful in real time.

## Abstract 2

A retrospective analysis of radiation damage behaviour in a statistically significant number of real-life datasets is presented, in order to gauge the importance of the complications not yet measured or rigorously evaluated in current experiments, and the challenges that remain before radiation damage can be considered a problem solved in practice.

## 1. Introduction

The source of most problems when measuring X-ray diffraction data from macromolecular crystals is that the data to be measured (diffraction) are systematically degraded by the only way to measure them (X-rays). The development and adoption of techniques to cryocool crystals (Teng, 1990 ▶; Garman & Schneider, 1997 ▶) extended crystal lifetimes by orders of magnitude; yet, as this was exploited to probe ever more weakly diffracting or smaller crystals with ever more intense and focused synchrotron beams, the problem of damage was merely postponed, albeit to a much higher dose limit, and the need to take crystal decay into account in practice has remained highly pertinent.

How to deal with radiation damage has attracted attention from the start (Blake & Phillips, 1962 ▶; Hendrickson, 1976 ▶), but over the last decade a directed research effort (Garman & Nave, 2009 ▶) has transformed our understanding of the phenomenon (Holton, 2009 ▶). Qualitatively, a distinction is made between specific damage, *i.e.* the disappearance of different groups of atoms at varying rates (Burmeister, 2000 ▶; Ravelli & McSweeney, 2000 ▶; Weik *et al.*, 2000 ▶); and global damage, which leads to the overall loss of diffraction of the crystal (Meents *et al.*, 2010 ▶; Nave & Hill, 2005 ▶; Warkentin & Thorne, 2010 ▶).

Of practical importance is that the progress of global damage has been rigorously quantified at 100 K, the commonly used cryogenic temperature, by relating the absorbed dose (deposited energy per mass unit) directly to its effect on the diffraction data. Once *RADDOSE* (Murray *et al.*, 2004 ▶) made it possible to estimate the dose, Owen *et al.* (2006 ▶) showed that the summed diffraction intensity decays in direct proportion to the absorbed dose, at resolution-dependent rates. Kmetko *et al.* (2006 ▶) showed that the resolution dependence could be described by the change in relative scaling *B* factor (Δ*B*_rel_), which changed in direct proportion to the absorbed dose. The proportionality coefficient, which they coined ‘coefficient of sensitivity to absorbed dose’ or *s*_AD_ and defined in units of mean-squared atomic displacement (*u* ^2^), was approximately the same for all four systems studied. Both studies observed the behaviour of a set of reference reflections, or the whole high-symmetry dataset, repeatedly measured in the course of decay.

Bourenkov *et al.* (2006 ▶) reported that linearity against dose was also present in Δ*B*_scal_ (our designation), namely the change in the *B* correction factor applied as part of internal scaling of datasets (Arnott & Wonacott, 1966 ▶) by the programs *HKL*2000 (Otwinowski *et al.*, 2003 ▶) and *SCALA* (Evans, 2006 ▶). This linearity was corroborated by Borek *et al.* (2010 ▶), and Bourenkov & Popov (2010 ▶) reported that it corresponded in magnitude and universality to *s*_AD_; when recast in the units reported by the scaling programs (*B* = 8π*u* ^2^), it takes the unexpectedly memorable form of 1 Å^2^ MGy^−1^ (hereafter: *B* sensitivity).

As pointed out by Borek *et al.* (2010 ▶), Δ*B*_scal_ is thus by far the most accessible proxy for dose in conventional data-collection experiments, when crystal lifetime is at a premium and careful controls are rare. Other metrics of damage progress include the *R*-based metrics *R*_d_ (Diederichs, 2006 ▶) and *R*_R_ (Borek *et al.*, 2007 ▶), but as the latter study also shows these do not directly quantify the dose and have not been shown to have the same universality.

Δ*B*_scal_ and *B* sensitivity provide the link between a crystal lifetime and the ‘resolution limit’ of the dataset. Howells *et al.* (2009 ▶) showed that at a given resolution the intensity will have halved after ∼ 10 MGy Å^−1^ of dose. Borek *et al.* (2010 ▶) mention a practical rule of thumb for the largest change in *B*_scal_ which still allows a given target resolution to be achieved: *B*_scal_ should stay two to four times below the initial resolution-squared. In contrast, the program *BEST* (Bourenkov & Popov, 2010 ▶) incorporates the crystal lifetime rigorously in calculating a full data-collection strategy for obtaining the maximum resolution from a crystal: an assumed linear Δ*B*_scal_, derived from the *B* sensitivity (1 Å^2^ MGy^−1^ by default), is combined with the dose rate that must be estimated with *RADDOSE*. The availability of these programs has allowed considerations of radiation damage to be made available to non-experts at beamlines, *e.g.* through implementation of its fully automated mode in the *EDNA* on-line data-analysis framework (Incardona *et al.*, 2009 ▶).

Δ*B*_scal_ as metric of dose does, however, suffer from an important complication: because its role is to correct for any resolution-dependent spot weakening throughout the whole dataset, it is also sensitive to both anisotropic diffraction and variations in dose across the volume of intersection of the crystal and beam. In the studies cited above, careful control was exercised over these non-trivial complications, not least because techniques to characterize either crystal or beam thoroughly enough are still at best highly experimental. Correspondingly, in the current versions of both *RADDOSE* and *BEST*, the crystal is assumed to be bathed in the beam. A third potential complication worth noting is that the existing studies have not characterized very weakly diffracting crystals that decay much faster than they yield sufficient diffracted photons for well measured data; while Δ*B*_scal_ is assumed to model this scenario as well, this has not yet been demonstrated.

To date, the effect of varying the intersection of beam and crystal on final data quality remains largely uncharacterized. This contrasts with the situation in practice, where the most important optimization in data collection is to match the beam and crystal size (Bourenkov & Popov, 2006 ▶); and at modern beamlines the beam is routinely smaller than the crystal anyway. Banumathi *et al.* (2004 ▶) and later Borek *et al.* (2007 ▶) proposed that the exact progress of the beam–crystal intersection would not significantly affect the linearity of the average dose over time, but only for crystals bathed in the beam and even then the proportionality constant would not be the same, as Holton (2009 ▶) made explicit in his mathematical treatment of the approximation. Indeed, our own attempts to optimize datasets from weakly diffracting crystals suggested that, as far as data quality was concerned, the effect could probably not be ignored; for instance, damaged crystal contributes only noise to the high-angle spots, so if measured simultaneously with undamaged crystal (in the course of crystal oscillation) it would deteriorate *I*/σ*I* and thus affect the overall resolution.

In this study we investigate the importance of these complications by assessing how well the decay behaviours in a statistically significant set of historical datasets, collected in real-life data situations without controls but optimized empirically for decay, conform to the expected linearity of Δ*B*_scal_ and *B* sensitivity of 1 Å^2^ MGy^−1^; whether the observed data quality could be accurately predicted automatically using the software tools now available; and whether both the discrepancies and the practicality of the analysis itself reveal systematic trends that need addressing before radiation damage can be considered robustly manageable when planning experiments in practice.

## 2. Methods

### 2.1. Samples and synchrotron

**Figure 1:**
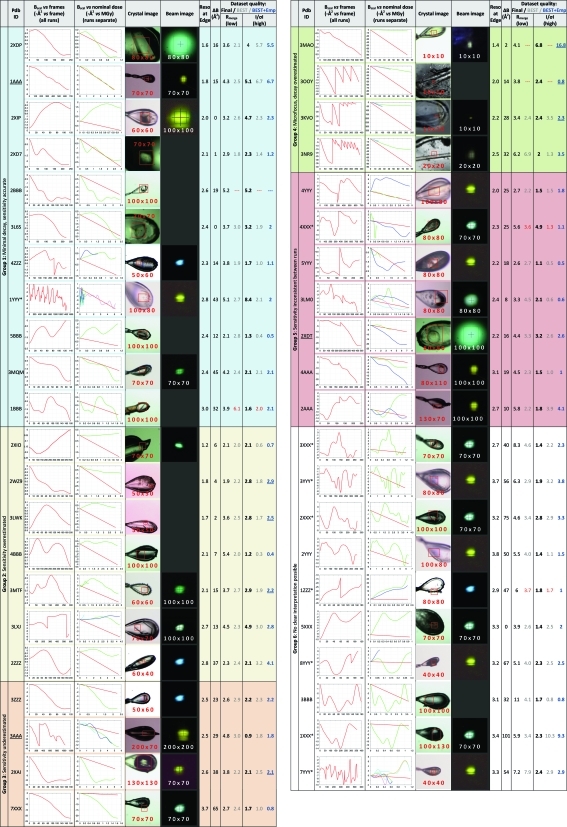
Collation of dataset information, decay statistics and data quality (actual and predicted) for the 43 datasets. The columns are described in §2.3. Background colours distinguish the overall categories (first column). The numbers on the images are the nominal beam size (beam slit settings, µm) used for the datasets (red numbers) and beam images (white numbers), respectively. (The beam structure seen in some of the beam images was addressed in subsequent beamline upgrades.)

The 43 datasets included in this report (Fig. 1 ▶) were collected from crystals of domains of 34 different human proteins generated at the Oxford site of the SGC (Gileadi *et al.*, 2007 ▶). These were collected over the course of 1 year (arbitrarily: September 2009 to August 2010): some comprise the best synchrotron datasets collected for the respective target proteins, 17 of which were recently deposited in the Protein Data Bank (PDB); the rest are a random selection collected during five specific visits, which are either not yet phased, did not achieve the target resolution of 2.8 Å required by SGC funding conditions or whose purpose was experimental phasing (marked in Figs. 1 ▶ and 2 ▶).

All data were collected on the macromolecular crystallography beamlines (I02, I03, I04 and I24) of the Diamond Light Source (Duke *et al.*, 2010 ▶; Evans *et al.*, 2007 ▶). Extensive metadata on each sample were available from the internal, manually curated laboratory database of the SGC (BeeHive^®^, Molsoft LLC); the metadata of data collection were available from the ISpyB database (Beteva *et al.*, 2006 ▶) that is automatically populated at Diamond; and extensive freeform notes were recorded during data collection in an electronic laboratory notebook (ConturELN^®^, Contur Software AB).

All datasets were collected from crystals selected after comprehensive pre-screening; crystals were mounted mostly in nylon loops (Hampton Research), as these allow crystals to be reoriented most easily for data collection. In a few cases crystals were mounted in LithoLoops (Molecular Dimensions Limited). At least two experienced crystallographers were usually available at the beamline for deciding data collection strategies.

### 2.2. Data collection strategy

The strategy goal for most of the datasets presented here was to maximize the resolution of the whole dataset, and attempt to achieve a final resolution equivalent to that estimated from initial test images. The remaining datasets were collected for experimental phasing (SAD), and the priority was to achieve high redundancy without decay. In all cases the beam size was matched to the crystal as closely as possible. The determination of the irradiation strategy represents an attempt to assimilate the findings from radiation damage research into our data collection protocols (compare Flot *et al.*, 2005 ▶), by empirically estimating the rate of decay for a particular crystal system on a beamline.

#### 2.2.1. Geometric strategy

*Geometric strategy* was calculated using *MOSFLM* (Leslie, 2006 ▶), to identify both a reasonable per-image oscillation to avoid spot overlap, and the smallest total rotation range to provide complete data, which Bourenkov & Popov (2006 ▶) showed to be the optimal strategy to combat detector readout noise. In a few cases, initial images could not be indexed within a reasonable time owing to severe lattice pathology; here a default strategy was used, namely a rotation range of 180° using 0.5° oscillations. For SAD datasets the default was to collect 360° of data.

#### 2.2.2. Irradiation strategy

*Irradiation strategy* was determined from an initial ‘exploratory dataset’, collected either from a poorer crystal or from one sub-volume of the best crystal, at an arbitrary but high dose rate. Data were processed simultaneously with data collection, by integrating with either *MOSFLM* (Leslie, 2006 ▶) or *XDS* (Kabsch, 2010 ▶) converting the *INTEGRATE.HKL* file to mtz format using *POINTLESS* (Evans, 2006 ▶) and scaling using *SCALA* (Evans, 2006 ▶). Crystal lifetime was measured as the number of seconds of unattenuated beam (total exposure time × beam transmission fraction) that either induced a decay in *B*_scal_ of 3–7 Å^2^, and/or loss of per-image resolution of 0.2 Å; these cut-offs were deliberately conservative. Δ*B*_scal_ was obtained from *SCALA* and per-image resolution was reported by *MOSFLM* as the highest resolution shell for which integrated *I*/σ*I* > 1. This subjective assessment relied heavily on intuition, factoring in anisotropy, crystal size, and the orientations of the loop and lattice. Where the data processing calculation failed, image resolution was gauged by eye from individual images throughout the dataset. The deduced lifetime was convoluted with the geometric strategy as described below. If the exploratory dataset had been collected from a sub-volume of the best crystal and the decay was judged sufficiently low, it was either used as the final dataset or further passes were collected with higher dose per oscillation. For SAD datasets the dose per image was set to yield a diffraction limit significantly lower than the limit of the crystal (Holton, 2009 ▶).

#### 2.2.3. Crystal partitioning strategy

The beam profiles at various slit settings were imaged with YAG (yttrium aluminium garnet) or BGO (bismuth germanate) screens available at the beamlines, with the beam transmission reduced so that the image was not saturated (images were not recorded for all sessions). For data collection, beam-size settings were selected which appeared to minimize the volume of non-crystal vitrified solution irradiated by the beam. If the selected beam dimension was smaller than the crystal in the direction of the rotation axis, all possible segments in that direction were tested for diffraction quality (Aishima *et al.*, 2010 ▶; Bowler *et al.*, 2010 ▶). The oscillation range determined from the geometric strategy was divided amongst the suitable segments, and the transmission and time-per-image were set so that each segment was irradiated for no more than the empirically determined lifetime, but maximizing transmission/image. Crystals were re-oriented if this increased the number of segments; given the absence of kappa goniometry, this was done manually by pushing with a sharpened pipette tip against the stem of the loop, with the experimenter crucially holding their breath so as not to disturb the stream of the cryostat.

#### 2.2.4. Software

With this approach it was vital that data are processed in real-time, and because only *MOSFLM* can be set to report explicitly the resolution limit of each image, close familiarity with this software was crucial, as were custom scripts. More recently, the deployment at Diamond of automatic data processing with *FASTDP* (Graeme Winter, personal communication) and extraction of per-image spot statistics with *LABELIT* (Sauter *et al.*, 2004 ▶) aided assessment considerably for routine cases, as did the modification of *POINTLESS* to enable processing of the output of *XDS* (Phil Evans, personal communication).

### 2.3. Retrospective analysis

The compilation of dataset information in Fig. 1 ▶ was generated as follows, with predictions calculated as automatically as possible in order to conform to the state of the art.

Dataset codes (column 2) are either the PDB ID of the deposited structure, or an arbitrary code comprising the data collection session and dataset number; SAD datasets are marked with asterisks. To generate the plot of *B*_scal_ *versus* all images (column 3), for all datasets, all frames were reintegrated with *XDS* (Kabsch, 2010 ▶); the unmerged data (in the file ‘*INTEGRATE.HKL*’) were converted to mtz format with *POINTLESS*, and all batches were scaled in *SCALA* as a single dataset with multiple runs, applying smooth scaling and the recommended absorption correction. (The exact scaling protocol, including the use or not of an absorption correction, had only a marginal effect on the values of *B*_scal_.) In the graph the frame number is plotted along the *x* axis and *B*_scal_ along the *y* axis (as a negative number, following the *SCALA* convention).

Crystal images (column 5) are those recorded automatically at the start of data collection. Images of the beam (column 6), where available, have been recorded at the start of each synchrotron visit and so do not always show the slit settings that were recorded for the corresponding datasets; they have been scaled in dimension to match the size of the crystal images. The resolution (column 7) is that at the edge of the detector, which in most cases corresponds approximately to the dataset resolution.

To place *B*_scal_ for each run on an absolute scale of nominal dose (column 4) and allow comparison of experimental *B* sensitivity, the dose rate was estimated using *RADDOSE* by making the same assumptions that an automated calculation would: that beams were top-hat shaped; that flux density remained identical to that of the reference flux at 0.1 mm × 0.1 mm, regardless of changes in beam area; that beam attenuation only scaled the intensity of the beam profile but not its shape; and that a 0.1 mm × 0.1 mm beam had a flux of, respectively, 4.6 × 10^11^, 10^12^ and 4.6 × 10^11^ photons s^−1^ on beamlines I02, I03 and I04 (values provided by beamline scientists). Thus, flux was calculated as (reference flux × transmission × actual area/reference area). For I24 the flux was taken as 8 × 10^11^ photons s^−1^ for all beam sizes, since here beam size is changed by refocusing the mirrors rather than cutting the beam. (The validity of these crucial assumptions is discussed below.) Crystal dimensions were estimated from the crystal snapshots, where necessary using the loop thickness (20 µm) as a reference; beam slit and attenuation settings were read from the ISpyB database; crystal composition was derived from data in the BeeHive database. For crystals containing soaked heavy atoms, in addition to the bound metal, we conservatively assumed a residual heavy-atom concentration of 1 m*M* in the solution after back-soaking (the heavy atoms were soaked at 10 m*M*). The relative dose for each frame was calculated as (frame number × flux-per-frame × time-per-frame × dose rate). For the graph for each run the dose was set to start from zero and *B*_scal_ was extracted from the same values plotted in column 3, but adjusted to start from zero (*i.e.* assuming no non-linear dose effects). All runs were plotted on the same graph and a line added corresponding to the *B* sensitivity of 1 Å^2^ MGy^−1^ used in *BEST*, as the internal reference. In the graphs *B*_scal_ is along the *y* axis (as in column 3) and the nominal dose along the *x* axis.

Dataset quality (columns 9–13) was assessed by two metrics: the signal-to-noise (*I*/σ*I*: ‘*Mn(I/sd)*’ in *SCALA*) in the highest shell reported by *BEST* (in our hands, always the edge of the detector); and *R*_merge_ in the lowest resolution shell. Final dataset statistics (columns 9 and 11) are those for merging the subset of frames and resolution that yielded the ‘best’ data, as judged subjectively by a crystallographer; in the case of deposited structures, these were the data used for refinement. This was also the dataset for which diffraction anisotropy (column 8) was calculated, using the Δ*B* reported by *PHASER* (McCoy *et al.*, 2007 ▶), which is (approximately) the difference between the *B* values along the most weakly and strongly diffracting directions in reciprocal space, respectively.

To obtain predictions of data quality achievable from each crystal (columns 10 and 12), *BEST* (version 3.2.0) was run using test images collected immediately preceding the dataset. These reference images were indexed with *LABELIT* and integrated with *MOSFLM* to provide suitable input files for *BEST*. The dose rate was that calculated by *RADDOSE* for the nominal dose (column 4). *BEST* was run using the same data collection parameters (oscillation start/end/range, exposure time/transmission) used to collect the images used in the observed data collection statistics (columns 9 and 11), to ensure that only dataset prediction was evaluated and not strategy calculation as well.

In order to evaluate the *BEST* prediction model independently of estimates of beam and crystal sizes, the predicted outer shell *I*/σ*I* (column 13) was recalculated by providing *BEST* with an observed dose rate, estimated by fitting a linear curve to Δ*B*_scal_ of the final dataset and converting it to a dose rate using the *B* sensitivity (1 Å^2^ MGy^−1^).

## 3. Results

### 3.1. Retrospective analysis: predictions and outcomes

Since our standard data collection procedure is highly customized and subjectively guided by experience and intuition, the dataset outcomes were compared (final dataset quality and decay statistics) to fully automated predictions using objective programs, namely *RADDOSE* and *BEST*, relying on automatically recorded experiment information. The comparison is shown in Fig. 1 ▶, where datasets have been grouped qualitatively according to the agreement between the decay used in *BEST* (*i.e. B* sensitivity: 1 Å^2^ MGy^−1^) and the nominal decay measured by Δ*B*_scal_ and the nominal dose (column 4):

(i) Group 1: Decay proceeds as expected or *B*_scal_ varies so little (<1 Å^2^) that no significant decay has occurred.

(ii) Group 2: Decay is severely overestimated; most crystals are far larger than the beam.

(iii) Group 3: Decay is severely underestimated; here the nominal beam size was set to match the crystal.

(iv) Group 4: These are the datasets collected with the microfocus beam (I24), which was smaller than the crystals (as in group 2); though significant decay is observed in all cases, the decay is very severely overestimated.

(v) Group 5: The multiple runs of each dataset do not show internally consistent decay; some runs do, however, agree with the estimated decay.

(vi) Group 6: *B*_scal_ behaviour is too non-linear to be interpretable as decay; in some cases the overall trends can be said to agree, but only qualitatively, owing to huge variations in *B*_scal_. This group contains most of the SAD datasets, so comparisons of predicted and observed resolution are less relevant.

**Figure 2:**
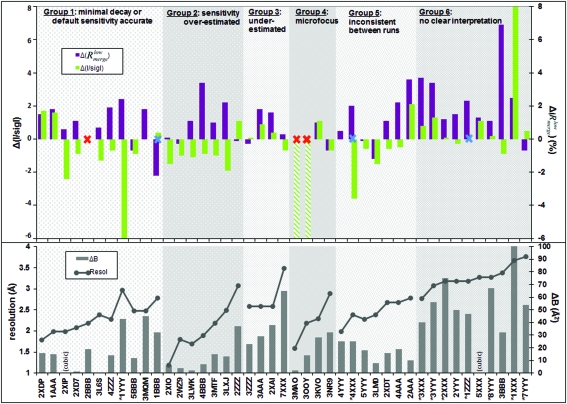
Discrepancies between actual and predicted dataset quality for the 43 datasets, juxtaposed with crystal properties. Datasets are ordered and labelled as in Fig. 1 ▶, and the plots are shaded to correspond to the six groups from that figure; within each group datasets are sorted by increasing resolution. Crystal properties are plotted in the lower panel: connected dots indicate dataset resolution and bars indicate anisotropy [Δ*B* of the final dataset as reported by *PHASER*; cubic crystals (indicated) are fully isotropic]. The upper panel plots the differences in the quality metrics (colours in the legend), with positive and negative corresponding, respectively, to over-optimistic and over-pessimistic estimates from *BEST*. The blue crosses indicate datasets where autoindexing failed, but which succeeded when restarted manually. The red crosses indicate where *BEST* prediction failed; the hatched columns (3MAO and 3OOY) represent very severe underestimates of the data in the outer shell (*R*_meas_ > 100%).

Fig. 2 ▶ shows the same grouping of datasets, but plotting a different set of criteria, namely the disagreement between observed and predicted data quality; this juxtaposition suggests a few trends which warrant further discussion below. A fully automated prediction could not be made in six cases (one in seven) because either autoindexing or the *BEST* prediction failed.

With *I*/σ*I* serving as the proxy for the resolution limit (a change of 1 unit corresponds to ∼0.1–0.2 Å), we note that this is under- and overestimated for groups 2 and 3, respectively, in accordance with the over- and underestimation of the decay rates. This trend extends to the microfocus datasets (group 4), where in two cases extremely high dose-rate estimates from *RADDOSE* led to *BEST* severely underestimating the resolution limits and therefore unable to calculate a strategy.

Most surprisingly, in group 1 where decay is well estimated, resolution is generally underestimated, sometimes severely. A possible explanation is that, because decay was generally low, weak high-resolution spots had more consistent counting statistics throughout the datasets and thus better average *I*/σ*I* than implied by the full decay assumed by the *BEST* model. If correct, this explanation highlights another problem with the general practice of citing ‘resolution’ as a proxy for dataset quality: namely that resolution is a function of an (arbitrary) *I*/σ*I* cut-off, a ratio which in turn is affected by very different effects, since it is reduced not only by data inconsistency and anisotropy but also by spot weakening through the dataset (decay), regardless of how well measured spots are to begin with. Borek *et al.* (2010 ▶) describe one approach to circumvent this, by first correcting data for weakening (decay) before scaling, but this has not yet been adopted in other scaling programs and thus was not tested here.

In contrast to resolution, according to another metric it can be seen that data quality is usually overestimated, namely 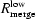, *i.e. R*_merge_ of only the lowest resolution shell data. The trend is evident across all groups of datasets; the differences observed are also rather large, given that we regard datasets with 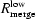 ≃ 10% with extreme suspicion. [This metric, based on the well measured data, is preferred to *R*_merge_ for all data, since for small values (*e.g.* weak measurements at high resolution) *R* metrics are mathematically unstable and not informative. Its use is philosophically similar to the approach described by Diederichs (2010 ▶).]

Groups 5 and 6 are relevant because here *B*_scal_ was *not* informative of the actual rate of decay. In group 6 this correlates with low resolution and very strong anisotropy for all (non-cubic) datasets: *B*_scal_ is known to be less well defined at lower resolution, and anisotropy is the other effect that had been factored out in previous decay studies (see §1). Thus, as expected, under these conditions (low resolution, high anisotropy), *B*_scal_ is a poor proxy for decay, certainly as determined by *SCALA*. For group 5, there is no clear trend to explain the behaviour.

What is striking is the prevalence of anisotropy (Fig. 2 ▶, lower panel) which is generally stronger at lower resolution. It does not, however, correlate with the (in-)accuracy of data quality predictions, so its disrupting effect remains hard to gauge. The use of observed decay rates was expected to improve predictions (*I*/σ*I* in column 13, Fig. 1 ▶) only for datasets in groups 2, 3 and 4, where the behaviour of *B*_scal_ was the most readily interpretable. In several cases (underlined in column 13) this was indeed the case, although many were not improved.

### 3.2. Case studies: unexpected sensitivity

As there is no reason to believe that any of the proteins in this study would have unexpected sensitivities, the many apparent deviations described in §3.1 are more readily explained by incorrect estimates of the nominal dose (the *x* axis in column 4, Fig. 1 ▶), in turn originating from inaccurate descriptions of the experiments passed to *RADDOSE*. Two case studies illustrate the problems.

**Figure 3:**
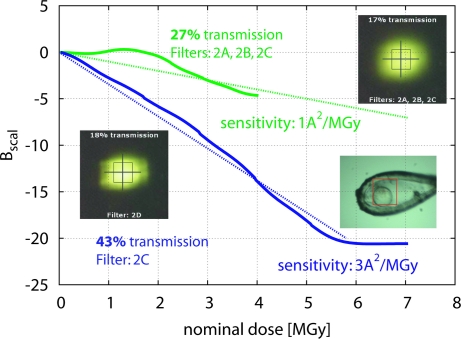
The importance of accurate characterization of the beam profile. Relative decay rates for the two runs of dataset 2XDT are shown as in Fig. 1 ▶, column 4 (description in §2.3), but recoloured for clarity; the dotted lines represent two different apparent sensitivity coefficients. Both runs were collected from the same crystal volume. Also shown are the beam images for almost equivalent attenuation, but achieved with different filters: inspection of the filter settings shows that filter 2A has a blurring effect which reduces the flux density inside the beam box by threefold, as judged by the sensitivity factors. (The blurring filter has since been inactivated at all beamlines.)

The effect of beam profile was evident in dataset 2XDT (group 5) which comprises two runs with apparently very different sensitivities, depending on the fluxes nominally determined by the beam filters (Fig. 3 ▶). Beam images taken at the start of the session indicated that one particular filter, that was also different between the two runs, caused a significant change of the apparent beam profile. Although the beam profile cannot be accurately characterized from these beam images because of saturated pixels, the particular filter appears to smear out the beam; thus, the centre of the beam would have a flux lower than expected, and the crystal, which was smaller than the nominal beam, will have absorbed a dose significantly lower than predicted by a factor of three, according to the change in apparent sensitivity.

**Figure 4:**
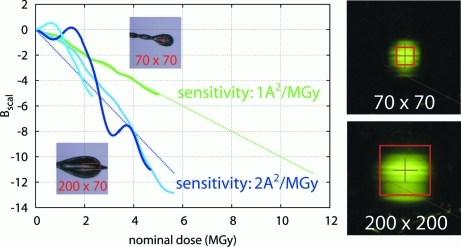
The importance of accurate characterization of beam dimensions. The decay rates of two datasets measured during the visit (green: 3AAA; blue: 1AAA) have been combined from Fig. 1 ▶ (column 4) for comparison. Both datasets were measured without filters but with different beam sizes (red boxes on inset crystal images). The dotted lines show the nominal sensitivities calculated assuming the same flux density for both beam settings (§2.3); it is, however, more likely that altered slit settings reduced the beam flux density in the box by a factor of two, a change not apparent in the beam images. Beam size labels are as in Fig. 1 ▶.

The effect of beam dimensions on beam profile was evident in the comparison of datasets 1AAA and 3AAA, both collected during the same session on the same beamline (Fig. 4 ▶), but with different slit settings in order to match the beam to the crystal. These crystals also show very different sensitivities, under the assumption that the beam flux scales proportionally only to the beam size (factor ∼2). It is more likely that the narrow slits setting had altered the beam-flux density profile unpredictably, or that the crystal for 1AAA was smaller than the nominal beam. What is relevant here is that these standard methods of characterizing both crystal and beam-flux density profile were insufficient to predict the decay rate accurately.

The very large discrepancies between the expected and observed decay in the microfocus datasets (group 4) is likely to be caused by similar errors, but amplified at such small dimensions: a 2–3 µm error is very significant for a beam nominally only 10 µm in diameter, and greatly alters the nominal flux density and thus dose rate.

### 3.3. Case study: generation of metrics

The reliability of obtaining the various metrics discussed here through different routes was investigated, using the apparently well behaved low-symmetry dataset 2XD7. Data were variously integrated and scaled with *MOSFLM/SCALA*, *XDS/SCALA* and *HKL*2000. In the case of *XDS*, data were converted with *POINTLESS* to mtz format suitable for input in *SCALA*, using unmerged data from *INTEGRATE* either before or after the *CORRECT* step of *XDS*.

**Figure 5:**
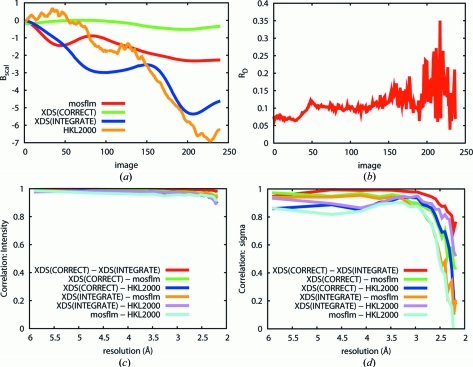
Variations in dataset and decay metrics from different programs, illustrated on dataset 2XD7. Labels correspond to the description in the main text. (*a*) Significant variations in *B*_scal_ are seen depending on which program was used. (*b*) *R*_d_ calculated by *XSCALE* (*XDS*) shows an upward trend, as expected, but does not help quantify the damage. (*c*) and (*d*) The correlation coefficient between the (*c*) intensities and (*d*) sigmas of intensities generated by different integration programs, plotted against resolution. While all programs extract similar intensities, they produce very different estimates of sigma for the high-resolution (weak) intensities.

Strikingly, *B*_scal_ does not agree for any two of the routes used (Fig. 5 ▶ *a*). Parenthetically, we note that deriving *B*_scal_ from data integrated with *XDS* is problematic: since *XDS* itself does not use *B*_scal_, unmerged data must be passed to *SCALA*, which does not, however, apply the Lorenz and polarization corrections missing from the unmerged integrated data (*INTEGRATE.HKL*). These can be added using *CORRECT*, but it also applies other basic scaling, even in non-scaling mode, that does significantly affect the values of *B*_scal_.

*R*-based metrics were not found to be more informative. *R*_d_, calculated with *XDS* and thus exactly as described in the literature (Diederichs, 2006 ▶), was noisy for this dataset (Fig. 5 ▶ *b*), presumably owing to the low multiplicity, as usual in the common low-symmetry space groups. Our attempt to calculate *R*_R_ as described by Borek *et al.* (2007 ▶) revealed that, in this low-symmetry case (monoclinic), the metric is as noisy as *R*_d_ (not shown).

Finally, given the importance of *I*/σ*I* for determining dataset resolution (and thus implied quality), the correlations of both *I* and σ*I* as determined by various routes (Figs. 5 ▶ *c* and 5*d*) were calculated. Astonishingly, although intensities are quite consistent between programs, the estimated values of σ*I* are widely different, as are the reported *I*/σ*I* at the same resolution [2.7, 3.5, 3.7 and 5.2 from *HKL*2000, *MOSFLM*, *XDS*(INTEGRATE) and *XDS*(CORRECT)]. While this observation is a familiar anecdote, it is also poorly understood; it is not lost on us that it complicates the comparison of predicted and observed data quality.

### 3.4. Generality of observations

Although all data were collected at a single synchrotron, we consider our observations to be general for two reasons. Firstly, Diamond beamlines correspond to state-of-the-art hardware and software available around the world, including (Duke *et al.*, 2010 ▶): stable beams that can be reduced below 100 µm × 100 µm with slits; high-resolution on-axis viewing cameras; large fast CCD detectors; variable-beam attenuation; (on beamline I24) a microfocus beam (<10 µm × 10 µm; Evans *et al.*, 2007 ▶) with a Pilatus P6M detector (Dectris Ltd, Baden, Switzerland). For the purposes of generalization, the presence or not of specific features available at selected beamlines elsewhere is thus not relevant.

Secondly, by the arguably only truly relevant metric, namely deposited structures, Diamond beamlines have allowed us to be as productive (2.8 datasets per structure, 4 structures per month) over the time period of these 43 datasets as we previously were over an equivalent time period using another state-of-the-art beamline, PXII of the Swiss Light Source (SLS) manuscript in preparation). SLS datasets were not included here because the absence of some metadata complicated the retrospective study.

## 4. Discussion

The analysis in §3.1 is intended as an assessment of the state of the art in radiation damage prediction, by comparing the fully automated predictions now conveniently possible against benchmark outcomes generated through far more laborious experiments. Our prediction calculations were therefore deliberately naive, ignoring obvious experimental features if not encoded, as this is a fundamental characteristic of automation: it corresponds to the high-throughput mode, where no analysis will be more accurate than the values reported by the hardware, and calculations that fail will result in experiments that are either unnecessarily aborted or else executed with default and suboptimal parameters. Not coincidentally, this also applies to inexperienced users.

Of course, users are generally more sophisticated, but effective methodology relieves all users of the need to think, so it is informative to consider these scenarios as a performance baseline in order to define the challenges that remain. We distinguish two classes of challenge: unresolved scientific questions; and how to make the solutions sufficiently accessible.

### 4.1. Scientific challenges

While the discrepancies in quality metrics plotted in Fig. 2 ▶ define the challenge, the crystal and beam images in Fig. 1 ▶ indicate clearly that a parameterization of the experiment consisting only of slit settings and transmission, as now recorded by beamlines and passed to *RADDOSE* as in our automated analysis, is wholly inaccurate: the crystals are rarely bathed in the beam, the beam flux-density profiles are neither top-hat nor Gaussian, and the crystals are not simple shapes. Fig. 2 ▶ also shows that anisotropy is not only common but systematically stronger at lower resolutions.

There may be two approaches to this challenge, although both require further confirmation. The first involves extending the existing approach of predicting the experiment from first principles, and appears to require a far more sophisticated characterization of the beam flux-density profile, the three-dimensional shape of the crystal and their relative orientation, all of which can be only very approximately extracted with the current routine techniques. Imaging the beam by scintillation on, for example, YAG is prone to over-interpretation, and knife-edge scans provide only projections of the convolution of the edge and the beam; to our knowledge, a satisfactory approach remains elusive. Direct imaging of crystals in the loop is also insufficient, being subject to severe refraction effects and providing no three-dimensional information; however, reports of the X-ray tomography of crystals (Brockhauser *et al.*, 2008 ▶) show significant promise. Such characterizations will link up with on-going developments of existing programs (*RADDOSE, BEST*) to make use of such information (*e.g.* Zeldin & Garman, private communication).

The second, empirical, approach would rely on exploratory datasets: these are quick to obtain, but their interpretation is not yet robust nor is using such information to predict final dataset quality and resolution. The interpretation may be simplified by protocols suggested even in the earliest days (Blake & Phillips, 1962 ▶), namely tracking *B*_rel_ by re-measuring specific reference images repeatedly through the course of the exploratory dataset; but such protocols are not routinely implemented on beamline hardware. Alternatively, if Δ*B*_scal_ is to remain the decay metric of choice, it would be important to have its relationship more precisely defined between the programs that make use of it, as well as establish how best to calculate it from programs that do not use it (*e.g. XDS*). Additionally, its applicability at resolutions worse than 3 Å needs to be rigorously established.

Which of the approaches, the *a priori* or the empirical, will be the more robust is not clear, although it is encouraging that using empirical dose rates leads to improved predictions for some of the group 2, 3 and 4 datasets (§3.1). The yardstick, however, will be the applicability to microfocus experiments (group 4) where the discrepancy between observation and prediction is particularly acute. Not only is the beam here routinely smaller (or much smaller) than the crystal, but these beamlines serve as the last resort for weakly diffracting (*e.g.* membrane protein) crystals, so that not only is strong anisotropy prevalent, but also the third complication discussed in §1, namely that the spots decay rapidly. The small beam also means that multi-segment datasets are common, so that weak images are spread through the dataset (‘saw-tooth’ *B*-factor plots, *e.g.* dataset 3OOY in Fig. 1 ▶) rather than appearing only towards the end; this too needs to be modelled.

The observation for group 1 datasets (§3.1) warrants further investigation, *viz.* that datasets with little decay yield better than predicted resolution: if verified, this would have significant implications for strategy, *e.g.* that multi-segment (even multi-crystal) datasets should become the norm rather than the exception. This would also suggest that the progression of the per-image resolution limit is a more robust predictor for dataset resolution; it has been informative in our hands, but this too needs to be rigorously demonstrated.

### 4.2. Logistic challenges

Equally important is the challenge of *accessibility* to diagnostics of radiation decay. What is most relevant about the analyses presented here is that they were available only retrospectively, for only a small fraction of our collection of over 1000 datasets, through the effort of many weeks and only after intense scrutiny of the arcana of the topic, an effort for which there is no time at the beamline. To be useful, tools must be available when and where needed, namely directly in conjunction with data collection so that it can be diagnosed and optimized immediately. Given how modern beams will destroy diffraction within seconds, it appears self-evident that the absence of such analyses must contribute substantially to the frequently cited low success rate of synchrotron datasets (1/50; Holton, 2005 ▶). In view of the scale of research funding reliant upon high-flux synchrotron beamlines, and the high and growing frequency of data collection experiments there, the problem would thus appear to be acute.

The general problem has indeed been approached through automated frameworks (*e.g. EDNA* or *Web-ICE*; González *et al.*, 2008 ▶), for which the ready-to-execute programs *RADDOSE* and *BEST* have been vital components. These are very important efforts, as they also address another major but more mundane problem, namely gathering and storing the data (*e.g. Web-ICE* or ISpyB), the sample information being a particular challenge since it resides with the user and obtaining it requires their cooperation, given the rarity of users with laboratory databases (LIMS).

Nevertheless, until the dimensions of the experiment can be thoroughly characterized and encoded, any attempts to automate fully both strategy and data collection using *a priori* approaches appear premature.

### 4.3. Beamline software and hardware

Addressing these challenges therefore requires a more, not less, interactive element in beamline software as more weakly diffracting crystals and stronger beamlines mean that data collection is becoming increasingly complicated. The relevant question is thus how to implement software tools so that they aid setting up a full experimental design, rather than how to substitute it with an automatically calculated strategy (*i.e.* rotation range, oscillation width, transmission), no matter how sophisticated. Three general features would be required in beamline software:

(i) A facility for sophisticated user interaction with the strategy, where the experiment is recast as primarily an intersection of a beam profile with a three-dimensional crystal, made concrete by an interactive graphical view with the (pre-calculated) strategy superimposed; where crystal volumes, orientations and other strategy assumptions can be specified, reviewed and modified; and where failed indexing calculations can be updated with manually corrected results.

(ii) A post-experiment analysis juxtaposing predicted and observed decay and quality of datasets; generating this must be consistently robust.

(iii) Making these facilities the default (and potentially non-optional) routes for setting up and reviewing data collections.

Such a software project may seem daunting, considering the number of components that must be integrated and the amount of additional experiment data that must be recorded. However, scientifically it appears far more achievable than fully reliable automation; and proper design can ensure that such features remain non-onerous when highly optimized data are not required (*e.g.* ligand complexes of well characterized crystals). At the same time, recasting the experiment as in (i) above raises the imperative to implement, for routine use, tools for characterization of both beam and crystal; a side-benefit would be that beamline problems will by definition be easier to identify, thanks to improved diagnostics.

Improving decay strategies will require hardware changes too, namely the ability to reorient crystals. Our willingness to do this manually (see §2.2) has frequently been not only beneficial by improving the visualization of the crystal but critical by allowing better partitioning of datasets over the crystal volume. Happily, such facilities have recently returned to fashion, *e.g.* the MiniKappa project at the ESRF beamlines (BIOXHIT, 2010 ▶).

## 5. Conclusions

Our observations suggest that the state-of-the-art for dealing with radiation damage in data collection, while impressive, does not yet account for a significant subset of real-life cases and there remain challenges for future developments:

(i) Robust ways to extract decay information from existing datasets to help plan future datasets; this might include deconvoluting *B*_scal_ from anisotropy, analysis of reference images, or the analysis of the loss of *I*/σ*I*.

(ii) Techniques to routinely characterize both beam profile and crystal volume, and software tools that can make use of the information, especially for multi-segment datasets.

(iii) Robust models for the use of microfocus beams.

(iv) Beamline software with integrated tools for highly interactive strategy and experiment design, and instant display of decay information.

(v) Making crystal-reorientation hardware commonplace.

In view of our experience that careful monitoring of radiation decay in real time can significantly improve the success rate of synchrotron datasets, we predict that investing in these points would boost community-wide productivity far more than building more beamlines or synchrotrons. Now that the underlying principles of radiation damage have been rigorously established, the time seems right to apply these to the more complex situations encountered in routine data collection, which would include selecting appropriately complex model systems.

We emphasize that the relevant question here is not whether any of these features are present at *specific* beamlines, or have been demonstrated before *in principle*; what matters is whether they have become *generally* established and by this criterion there is no doubt that the challenge is far from met.
